# Temporal and geographic variation in the systemic treatment of advanced prostate cancer

**DOI:** 10.1186/s12885-018-4166-3

**Published:** 2018-03-06

**Authors:** Megan E. V. Caram, Jason P. Estes, Jennifer J. Griggs, Paul Lin, Bhramar Mukherjee

**Affiliations:** 1 Department of Internal Medicine, Division of Hematology/Oncology, University of Michigan Medical School, Ann Arbor, MI USA; 2 Ann Arbor Veterans Affairs Healthcare System, Ann Arbor, MI USA; 3 Institute for Health Policy and Innovation, University of Michigan, Ann Arbor, MI USA; 4 Department of Biostatistics, School of Public Health, University of Michigan, Ann Arbor, MI USA; 5 Department of Health Management and Policy, School of Public Health, University of Michigan, Ann Arbor, MI USA

**Keywords:** Prostate cancer, Novel agents, Variation, Care delivery, Claims data

## Abstract

**Background:**

Several systemic treatments have been shown to increase survival for patients with metastatic castration-resistant prostate cancer. This study sought to characterize variation in use of the six “focus drugs” (docetaxel, abiraterone, enzalutamide, sipuleucel-T, radium-223, and cabazitaxel) that have been approved by the Food and Drug Administration for the treatment of metastatic castration-resistant prostate cancer during the years 2010–2015. We hypothesized that the use of these treatments would vary over time and by region of the country.

**Methods:**

We used Clinformatics DataMart™ Database (OptumInsight, Eden Prairie, MN), a de-identified claims database from a national insurance provider. Our sample included patients with prostate cancer who received any of the six drugs. We describe changes in usage patterns over time and geographic region of the United States via detailed descriptive statistics. We explore both patterns of first line therapy and sequence of treatments in our database.

**Results:**

Our final analysis included 4275 patients with a mean age of 74 years. Docetaxel was the most commonly used first-line therapy in 2010 (97%), 2011 (66%), and 2012 (49%). Abiraterone was the most commonly used first-line therapy in 2013 (56%), 2014 (46%), and 2015 (34%). Approximately 14% of our study cohort received ≥3 of the 6 drugs throughout their disease course. There was marked geographic variation in use of each of the drugs.

**Conclusion:**

Variation in treatment patterns were found with respect to both time and geographic location. Prescription rates of abiraterone outpaced docetaxel as the most commonly prescribed drug after 2013 when it became widely available. However, some regions of the country still lagged behind and prescribed less than would be expected.

**Electronic supplementary material:**

The online version of this article (10.1186/s12885-018-4166-3) contains supplementary material, which is available to authorized users.

## Background

Prostate cancer, one of the leading causes of cancer death in men in the United States, is treated with androgen deprivation therapy (ADT) in its advanced form of metastatic disease [1]. Most patients with metastatic prostate cancer will require additional treatments beyond ADT when their cancer progresses to castration-resistant disease, the lethal form of prostate cancer. The Food & Drug Administration (FDA) approved docetaxel in 2004 for the treatment of patients with metastatic castration-resistant prostate cancer (mCRPC) based on its ability to improve overall survival [2]. Since 2010, five new therapies have been FDA-approved to treat patients with mCRPC. Sipuleucel-T and cabazitaxel were each approved in 2010 [3, 4]. Abiraterone and enzalutamide were approved in 2011 and 2012, respectively, [5–8] and most recently, radium-223 was approved in 2013 after showing an improvement in overall survival in men with symptomatic bone metastases [9].

Due to the rapid emergence of these new therapies, which we refer to as our “focus drugs,” little is known about their adoption since approval and there is limited guidance on their use. Importantly, with the exception of docetaxel, the average wholesale price for each of these medications is greater than $8000 a month and insurance coverage for these treatments are included under different forms of payment structures within payer programs. Abiraterone and enzalutamide are relatively well tolerated oral medications and are typically covered through prescription drug plans with patient co-payments and without direct office reimbursements. Docetaxel and cabazitaxel are associated with greater side effects and are therefore more difficult for some patients to tolerate but are covered in a different part of the health plan and may cost less to patients out of pocket due to lack of co-payments. Similarly, radium-223 and sipuleucel-T are not chemotherapy agents but are delivered intravenously and require specialized centers to administer. Therefore, use is likely to be influenced by whether a practice invested in the infrastructure needed to deliver these specialty medications (for example, radium-223 requires existence of a nuclear medicine facility).

To better understand the real-world use of these treatments, we sought to characterize initial treatment patterns of the six focus drugs subsequent to FDA-approval in a large sample of patients with prostate cancer covered by a national private insurance provider. We expected that adoption of all of the focus drugs, except docetaxel, would gradually increase with time after FDA-approval and that the use of docetaxel would consequently decline as the newer agents were substituted for docetaxel. We expected that the oral medications (abiraterone and enzalutamide) would gradually outpace intravenous medications despite the decreased revenue to practices since they can be more widely prescribed by physicians and allow some physicians without access to infusion centers or tertiary care centers to keep their patients for longer. However, we expected to see geographical differences in adoption of the newer agents with a preference in rural communities for oral therapies due to difficulty in frequent travel required for intravenous chemotherapy. We also hypothesized that many patients would be prescribed more than one drug at a time despite the lack of evidence for benefit for combination therapy. We use the claims database of a large national insurance provider to explore these hypotheses.

## Methods

### Data source

Data for this study was obtained from the Clinformatics Data Mart (CDM), a de-identified database (OptumInsight, Eden Prairie, MN) from a large, national US health insurer. This database includes administrative health claims data of approximately 61 million unique patients across the United States, including enrollment records, medical claims, and prescription claims, from 2001 to the latest data available (currently June 2015). Among patients 65 years of age and older, three quarters of patients in the OptumInsight database have Medicare Advantage plans while the rest are enrolled through private insurance plans. The database is updated every six to twelve months, and each member is assigned one unique identification number regardless of a break in coverage.

### Participants and sample selection

To identify patients with prostate cancer in the OptumInsight Claims Database (OCD), the diagnosis fields (DIAG1 – DIAG5) within the medical claims data were matched against 185, the International Statistical Classification of Diseases and Related Health Problems (ICD) 9-CM for a prostate cancer diagnosis. Patients with a 185 diagnosis during the study period of January 1, 2010 to June 30, 2015 who received one of the six focus drugs were included. To identify patients who received any of the focus drugs, we matched national drug codes (NDC), brand names and Healthcare Common Procedure Coding System (HCPCS) codes associated with the six focus drugs against appropriate fields within the medical claims and pharmacy claims data. Patient demographic variables such as age, race, geographic location, and education level are available by matching on patient identification numbers across data files. In order to be considered for analysis of drug treatment patterns and in order to identify “first drug” for patients with advanced prostate cancer, we further required continuous enrollment of 180 days prior to receipt of one of the focus drugs (abiraterone, enzalutamide, docetaxel, cabazitaxel, sipuleucel-T, or radium-223), with no claim for one of the six drugs during that 180 days. Continuous enrollment was defined as less than 30 days break in coverage. In order to identify patients who had metastatic disease, the following ICD-9-CM codes were included: 196.x, 197.x, 198.x, 198.80, 198.81, 198.82, and 198.89. We refer the reader to the Additional file 1: supplementary materials for a detailed description of our sample selection methods.

### Statistical analysis

For each patient in our cohort, we identified pharmacy and medical claims for the six focus drugs over follow-up periods. Among these claims, we defined first-line therapy for each patient as the focus drug associated with the first claim with respect to time. We construct line plots of therapy administration rates and first-line therapy administration rates stratified by year to observe temporal patterns in treatment. In addition, we calculate 95% confidence intervals (based on the Wilson estimate) for the first-line therapy administration rates by year and tabulate the claims by therapy and year. To test for geographical variation in treatment, we carried out a chi-squared test of independence between first-line of therapy and geographical region.

To identify patients who received more than one drug concurrently, we implemented a search algorithm to find therapy sequences containing at least two overlapping sequences that met the following criteria: a claim for a focus drug is registered between two claims for a different focus drug, the difference between the two claims being no more than five weeks apart. We carried out a sensitivity analysis to the choice of “five weeks” as a time window to assess the robustness of our findings.

## Results

We identified 295,525 patients with prostate cancer over the study period January 1, 2010 to June 30, 2015 and 5659 patients who received one of the six focus drugs during that time. The final study cohort of patients included 4275 patients who were continuously enrolled for 180 days before receipt of first focus drug. The mean age of patients was 74 (standard deviation, S.D. 9). Further descriptive characteristics can be found in Table 1. All variables were significantly different in distribution (*p* < 0.05) between the larger prostate cancer sample and our selected sample of patients who received any one of the focus drugs. Age, education level, household income, region, insurance product, and presence of metastatic disease were strongly significant (*p* < 0.001) due to the large sample size. However, observed differences among some of these variables across the two groups may not be practically relevant.

**Table 1:** Patient Characteristics

	*n* = 4275	*N* = 295,525
Variable	Mean ± SD/Count (%)	Mean ± SD/Count (%)
Age	73.7 ± 9.2	71.1 ± 9.8
Race		
White	3001 (70.2)	208,702 (70.6)
Black	553 (12.9)	33,282 (11.3)
Hispanic	324 (7.6)	23,328 (7.9)
Asian	84 (2.0)	6144 (2.1)
Unknown	313 (7.3)	24,069 (8.1)
Education level		
Less than 12th grade	32 (0.7)	1681 (0.6)
High school diploma	1223 (28.6)	82,070 (27.8)
Less than bachelor degree	2238 (52.4)	153,094 (51.8)
Bachelor degree plus	599 (14.0)	42,997 (14.5)
Unknown	183 (4.3)	15,683 (5.3)
Household income range		
< 50 k	1544 (36.1)	93,629 (31.7)
50 k-99 k	1312 (30.7)	90,934 (30.8)
> 100 k	837 (19.6)	70,373 (23.8)
Unknown	582 (13.6)	40,589 (13.7)
Geographic Region*		
New England	203 (4.7)	15,488 (5.2)
Middle Atlantic	299 (7.0)	25,371 (8.6)
South Atlantic	950 (22.2)	71,420 (24.2)
East North Central	567 (13.3)	38,633 (13.1)
East South Central	131 (3.1)	9677 (3.3)
West North Central	448 (10.5)	27,567 (9.3)
West South Central	421 (9.8)	27,438 (9.3)
Mountain	441 (10.3)	27,673 (9.4)
Pacific	628 (14.7)	35,722 (12.1)
Unknown	187 (4.4)	16,536 (5.6)
Product		
HMO	2079 (48.6)	121,353 (41.1)
PPO	547 (12.8)	39,180 (13.3)
Other	1649 (38.6)	134,955 (45.7)
Unknown	0 (0.0)	37 (0.0)
Metastatic		
Yes	3446 (80.6)	17,802 (6.0)
No	829 (19.4)	277,723 (94.0)

SD, standard deviation; k, one thousand dollars; HMO, health maintenance organization; PPO, preferred provider organization; ASO, administrative services only (self-funded health plan)

*States within each geographic region:

• New England: Connecticut, Maine, New Hampshire, Rhode Island, Vermont, Massachusetts

• Middle Atlantic: New Jersey, New York, Pennsylvania

• South Atlantic: Delaware, Washington D.C., Florida, Georgia, Maryland, North Carolina, South Carolina, Virginia, West Virginia

• East North Central: Illinois, Indiana, Michigan, Ohio, Wisconsin

• East South Central: Alabama, Kentucky, Mississippi, Tennessee

• West North Central: Iowa, Kansas, Minnesota, Missouri, Nebraska, North Dakota, South Dakota

• West South Central: Arkansas, Louisiana, Oklahoma, Texas

• Mountain: Arizona, Colorado, Idaho, Montana, Nevada, New Mexico, Utah, Wyoming

• Pacific: Alaska, California, Hawaii, Oregon, Washington

Study cohort (*n* = 4275) and all patients with prostate cancer (*N* = 295,525) over the study period. Baseline characteristics for the study cohort were observed at time of initiation of first-line therapy. Baseline characteristics for the cohort of all patients with prostate cancer were observed at time of first medical claim for prostate cancer during the study period. Patients were classified as metastatic if a medical claim with ICD-9 codes 196.x, 197.x, 198.x, 198.80, 198.81, 198.82, and 198.89 were observed at any point during follow up

**Fig. 1:**
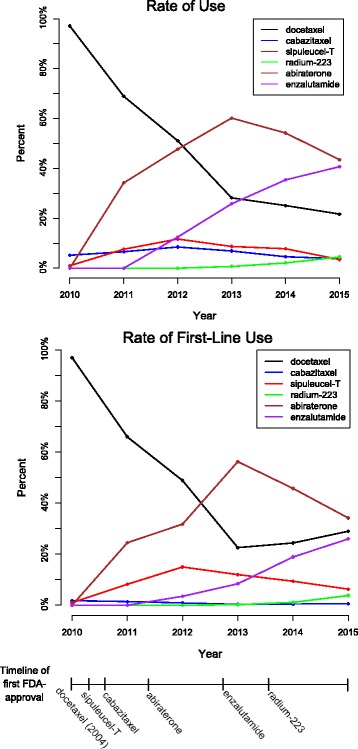
Title. Rates of Therapy Use. The top panel shows administration rates of the different therapies over years 2010 through mid-2015, defined as the proportion of patients who received that drug at some point in that year out of all patients who received any of those drugs that year. Rates were calculated for each drug for each year. The bottom panel shows administration rates of first-line therapy among the same cohort of patients, defined as the proportion of patients who received the drug as first-line therapy out of all patients who received a drug in that year. Administration rates of first-line therapy should sum to 100% in each year. The timeline below the two plots illustrates the approximate date at which each treatment was first approved for use in metastatic castration-resistant prostate cancer by the Food and Drug Administration (FDA). Docetaxel was approved in 2004 which is earlier than the timeline can demonstrate, but the other five treatments have a mark at the approximate point in time they were first approved

Figure 1 illustrates a time indexed plot of the proportion of patients in our sample that received each drug as first-line therapy in a given year and the most commonly prescribed first-line drugs. Docetaxel was the most common first-line therapy in 2010 (97.1%), 2011 (66.0%), and 2012 (48.9%). Abiraterone was the second most common first-line therapy in 2011 (24.5%) and 2012 (31.8%), but became the most commonly prescribed first-line therapy in 2013 (56.3%), 2014 (45.8%), and 2015 (34.2%). In contrast to the oral agents, abiraterone and enzalutamide, the usage of sipuleucel-T did not rapidly increase over time despite FDA-approval in 2010. Details for each drug frequency can be found in Table 2.

**Table 2:** Frequency distribution of first-line therapy

Therapy/Year	2010	2011	2012	2013	2014	2015	All
Docetaxel	374	434	391	207	255	137	1798
	97.1%	66.0%	48.9%	22.6%	24.4%	29.0%	42.1%
	(95.0, 98.4)	(62.3, 69.5)	(45.4, 52.3)	(20.1, 25.5)	(21.9, 27.1)	(25.1, 33.2)	(40.6, 43.5)
Cabazitaxel	7	9	7	3	4	3	33
	1.8%	1.4%	0.9%	0.3%	0.4%	0.6%	0.8%
	(0.9, 3.7)	(0.7, 2.6)	(0.4, 1.8)	(0.1, 1.0)	(0.1, 1.0)	(0.2, 1.8)	(0.6, 1.1)
Sipuleucel-T	4	54	120	110	98	30	416
	1.0%	8.2%	15.0%	12.0%	9.4%	6.3%	9.7%
	(0.4, 2.6)	(6.3, 10.6)	(2.7, 17.6)	(10.1, 14.3)	(7.8, 11.3)	(4.5, 8.9)	(8.9, 10.7)
Radium-223	0	0	0	2	12	18	32
	0.0%	0.0%	0.0%	0.2%	1.1%	3.8%	0.7%
	(0.0, 1.0)	(0.0, 0.6)	(0.0, 0.5)	(0.1, 0.8)	(0.7, 2.0)	(2.4, 5.9)	(0.5, 1.1)
Abiraterone	0	161	254	515	479	162	1571
	0.0%	24.5%	31.8%	56.3%	45.8%	34.2%	36.7%
	(0.0, 1.0)	(21.3, 27.9)	(28.6, 35.1)	(53.1, 59.5)	(42.8, 48.9)	(30.1, 38.6)	(35.3, 38.2)
Enzalutamide	0	0	28	77	197	123	425
	0.0%	0.0%	3.5%	8.4%	18.9%	26.0%	9.9%
	(0.0, 1.0)	(0.0, 0.6)	(2.4, 5.0)	(6.8, 10.4)	(16.6, 21.3)	(22.3, 30.1)	(9.1, 10.9)
**Total**	385	658	800	914	1045	473	4275

Frequency distribution of first-line therapy among 4275 patients who have a diagnosis of prostate cancer and received one of the listed focus drugs during the study period, stratified by year. Column percents and 95% confidence intervals (in parentheses) are also provided. Of note, data from 2010 to 2014 include the entire year, but data from 2015 is for only six months, January 1, 2014 through June 30, 2015

**Fig. 2:**
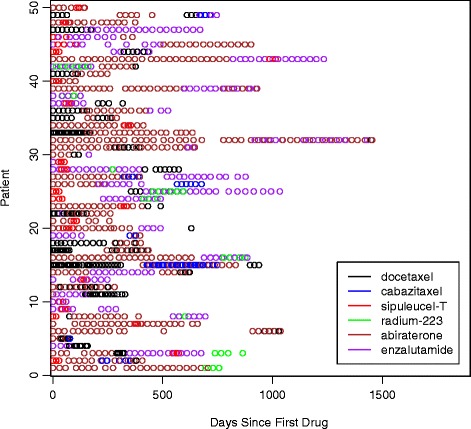
Treatment Patterns. Treatment patterns for 50 randomly selected patients who were identified to have received at least two distinct therapies concurrently during follow-up. Each horizontal row of circles represents one patient and each colored circle represents a claim for a drug for that patient. For example, the first patient listed at the top of the figure appears to have received 7 claims for abiraterone over a period of several months, but also received three claims for sipuleucel-T at some point during the time they were also undergoing treatment with abiraterone

We were able to determine the total number of distinct focus drugs that a patient received throughout their course of treatment for patients who started their first treatment in a given year. In 2011, 57.3% (*n* = 377) of patients received one therapy over the course of treatment and 42.7% (*n* = 281) received at least two. The proportion of patients receiving more than one drug throughout their course of treatment increased for those patients who started a drug in 2012; 50.4% (*n* = 403) of patients who started a treatment in 2012 received one therapy over the course of treatment, 48.6% (*n* = 397) went on to receive more than one focus drug during treatment and 21.4% (*n* = 170) received at least three. The majority of these drugs were given sequentially and not concurrently. We only report the frequencies for 2011 and 2012 since follow-up is shorter for patients who started therapy after 2012. There were 88 patients identified who received more than one of the six focus drugs concurrently; Fig. 2 illustrates claims patterns for 50 randomly selected patients who received more than one of the six focus drugs concurrently during follow-up. The most common combination was docetaxel plus either abiraterone (19%) or enzalutamide (10%). Sipuleucel-T plus either abiraterone (10%) or enzalutamide (11%) was also seen as well as radium-223 plus abiraterone (8%) or enzalutamide (13%). We also observed two patients who received three drugs concurrently (one patient received abiraterone, enzalutamide, and docetaxel concurrently; one patient received abiraterone, enzalutamide, and cabazitaxel concurrently).

**Fig. 3:**
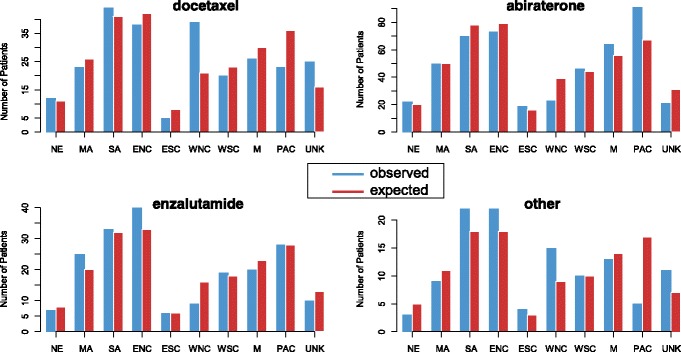
Observed versus expected use of therapies by region. Observed number of patients (blue) who received the indicated drug as first-line of therapy and number of patients expected (red) to have received the drug as first-line, by geographic division, in 2014. Expected numbers are calculated under the assumption of independence between geographic division and focus drug and account for the fact that there are differing numbers of patients enrolled in different regions. The category “Other” includes sipuleucel-T, radium-223, and cabazitaxel. NE, New England; MA, Middle Atlantic; SA, South Atlantic; ENC, East North Central; WNC, West North Central; WSC, West South Central; M, Mountain; PAC, Pacific; UNK, Unknown. States within each geographic region: New England: Connecticut, Maine, New Hampshire, Rhode Island, Vermont, Massachusetts. Middle Atlantic: New Jersey, New York, Pennsylvania. South Atlantic: Delaware, Washington D.C., Florida, Georgia, Maryland, North Carolina, South Carolina, Virginia, West Virginia. East North Central: Illinois, Indiana, Michigan, Ohio, Wisconsin. East South Central: Alabama, Kentucky, Mississippi, Tennessee. West North Central: Iowa, Kansas, Minnesota, Missouri, Nebraska, North Dakota, South Dakota. West South Central: Arkansas, Louisiana, Oklahoma, and Texas. Mountain: Arizona, Colorado, Idaho, Montana, Nevada, New Mexico, Utah, Wyoming. Pacific: Alaska, California, Hawaii, Oregon, Washington

We were able to demonstrate administration rates of the focus drugs as first-line therapy by geographic division in 2014, the first year that all focus drugs were FDA-approved for their most updated indication. These rates are shown in Table 3. Figure 3 illustrates the expected versus the observed counts under the assumption of independence between therapy and geographic division. The calculation of the expected frequency takes into account the distribution of patients included across different geographical regions. A chi-squared test yielded a *p*-value < 0.001, indicating evidence supporting a difference in administration rates across regions. For example, the average rate of use for abiraterone as a first-line therapy in 2014 was 45.8%. However, among patients in our cohort belonging to the Pacific division (AK, CA, HI, OR and WA), 61.9% of patients received abiraterone as a first-line therapy in 2014 in contrast to 26.7% of patients belonging to the West North Central division (IA, KS, MN, MO, NE, ND, and SD), indicating significant geographic regional differences in preference for first-line therapy in 2014.

**Table 3:** Observed versus expected receipt of focus drug in 2014

Region	Docetaxel (%)	Cabazitaxel (%)	Sipuleucel-T (%)	Radium-223 (%)	Abiraterone (%)	Enzalutamide (%)	Row Total
New England	12 (27.3)	0 (0.0)	3 (6.8)	0 (0.0)	22 (50.0)	7 (15.9)	44
	11	0	4	1	20	8	
Middle Atlantic	23 (21.5)	0 (0.0)	6 (5.6)	3 (2.8)	50 (46.7)	25 (23.4)	107
	26	0	10	1	50	20	
South Atlantic	44 (26.0)	0 (0.0)	20 (11.8)	2 (1.2)	70 (41.4)	33 (19.5)	169
	41	0	16	2	78	32	
East North Central	38 (22.0)	3 (1.7)	17 (9.8)	2 (1.2)	73 (42.2)	40 (23.1)	173
	42	0	16	2	79	33	
East South Central	5 (14.7)	0 (0.0)	4 (11.8)	0 (0.0)	19 (55.9)	6 (17.6)	34
	8	0	3	0	16	6	
West North Central	39 (45.3)	0 (0.0)	12 (14.0)	3 (3.5)	23 (26.7)	9 (10.5)	86
	21	0	8	1	39	16	
West South Central	20 (21.1)	0 (0.0)	9 (9.5)	1 (1.1)	46 (48.4)	19 (20.0)	95
	23	0	9	1	44	18	
Mountain	26 (21.1)	0 (0.0)	13 (10.6)	0 (0.0)	64 (52.0)	20 (16.3)	123
	30	1	12	1	56	23	
Pacific	23 (15.6)	0 (0.0)	5 (3.4)	0 (0.0)	91 (61.9)	28 (19.0)	147
	36	1	14	2	67	28	
Unknown	25 (37.3)	1 (1.5)	9 (13.4)	1 (1.5)	21 (31.3)	10 (14.9)	67
	16	0	6	1	31	13	
Column Total	255	4	98	12	479	197	1045

States within each geographic region:

• New England: Connecticut, Maine, New Hampshire, Rhode Island, Vermont, Massachusetts

• Middle Atlantic: New Jersey, New York, Pennsylvania

• South Atlantic: Delaware, Washington D.C., Florida, Georgia, Maryland, North Carolina, South Carolina, Virginia, West Virginia

• East North Central: Illinois, Indiana, Michigan, Ohio, Wisconsin

• East South Central: Alabama, Kentucky, Mississippi, Tennessee

• West North Central: Iowa, Kansas, Minnesota, Missouri, Nebraska, North Dakota, South Dakota

• West South Central: Arkansas, Louisiana, Oklahoma, and Texas

• Mountain: Arizona, Colorado, Idaho, Montana, Nevada, New Mexico, Utah, Wyoming

• Pacific: Alaska, California, Hawaii, Oregon, Washington

Number of patients (percent) in our study cohort who received the indicated focus drug as first-line therapy by geographic region in 2014 on the top line and number of patients expected to have received the indicated focus drug as first-line of therapy under the assumption of independence between geographic region and focus drug are also provided on the second line of each cell

## Discussion

This study found that the most common treatment prescribed between 2010 and 2015 among the six novel agents for advanced prostate cancer was abiraterone. Notable temporal patterns emerged throughout the observed period, including the declining preference of docetaxel as first-line treatment over time, likely a consequence with the approval of abiraterone in 2011, and a decline of abiraterone use when enzalutamide was FDA-approved toward the end of 2012. These patterns indicate a potential substitution effect of abiraterone for docetaxel and later enzalutamide for abiraterone. We would expect with longer times on the market, the frequency of use of abiraterone and enzalutamide may begin to mirror the therapy that is preferred by patients and providers as first-line treatment rather than being driven by the timing of FDA-approval of treatments. Even though the adoption of the oral agents increased with time, as we predicted, we were surprised that the use of sipuleucel-T and radium-223 did not increase the longer they were available despite clinical trials showing excellent tolerance of therapy. This could be due to the more complicated administration of those treatments. Another observation we made was that there was a substantial number of patients we identified who were prescribed more than one focus drug concurrently, a practice that is not evidence-based and may be associated with unexpected side effects as well as high costs. What is perhaps more unsettling is that our observation of frequency of concurrent use is likely to be an underestimation of the actual frequency of this practice considering our strict criteria for identifying concurrent use of therapies. Finally, we observed significant variation in regards to adoption of these agents by region of the country. Some regions appeared to favor intravenous agents and some the oral agents as first-line treatment. There were also regions of the country that used a higher than expected rate of sipuleucel-T and radium-223 while others used lower than expected rates.

This the first study to our knowledge that describes the contemporary treatment patterns for advanced prostate cancer and includes all six focus drugs in a large cohort of patients. Most standard clinical trials do not investigate drug sequences within the same trial but tend to focus on one medication. Large observational studies done with large databases allow the investigation of several patterns of drugs manufactured by different pharmaceutical companies in concert. This enables comparison of not just one drug but of the entire drug sequence and in the future, potential outcomes corresponding to those different drug sequences. Some studies have shown emerging practice patterns for systemic therapy in patients with mCRPC, such as medical oncologists prescribing more oral therapy and chemotherapy than urologists, [10] and evidence that prescriptions for abiraterone and enzalutamide vary geographically across the United States [11]. However, comparable studies demonstrating variation among all six focus drugs and relative variation in large datasets are lacking. While the temporal variation that we observed was somewhat expected as the use of a drug increases from the time a drug is introduced, the rate of change was not the same for each treatment. Moreover, there are no data in the literature with concrete evidence of the temporal changes. The geographic variation was also important to illustrate. Geography is a non-clinical factor that has been shown in the literature to influence variation in health care practices in several conditions, including treatment of back pain, [12] tonsillectomy, [13] and other surgical and cardiac procedures, [14] but has not been described in the systemic treatment of advanced prostate cancer. While geographic variation may be an independent contributor to variation, geographic variation in treatment patterns may be a result of other factors associated with geography, such as rural/urban differences, patient income, race, and health system factors.

Another potential contributor to geographic variation that has not been well-described in prostate cancer treatment may result from physicians and hospitals. Physicians from different specialties (e.g. medical oncology and urology) treat patients with advanced prostate cancer, and therefore different guidelines and national organizations are involved in guideline recommendations [15, 16]. Physician practices may get reimbursed higher for intravenous chemotherapy as opposed to oral medications that are dispensed through pharmacies, and whether a practice has invested in the infrastructure necessary to deliver specialty medications such as sipuleucel-T or radium-223 is likely to affect referral for these two treatments. Literature has shown that urology practices invested in radiation technology are more likely to treat patients with radiation than practices that do not, even in patients with a low likelihood of benefit from definitive treatment [17]. In addition, considering the high cost of treatment options for mCRPC, financial considerations for the patient become important factors as well. Some patients have tremendous out of pocket expenditures for certain treatment options that can lead to significant financial toxicity, despite the fact that they may have insurance coverage [18–20]. Except for docetaxel, each of the studied agents has an average wholesale price of at least $8000 a month and different insurance plans have varying coverage and co-pays for medications that are administered intravenously versus oral drugs. Thus, the out-of-pocket costs for oral drugs may be substantially higher than that for chemotherapy and thus income level is likely to factor in to treatment decisions. We expect to further evaluate these important non-clinical variables and their influence on treatment patterns in future studies. However, the most important first step was to define the landscape of treatment patterns adopted over time.

Due to the nature of observational research, there were limitations inherent to analyzing data using a large medical claims database such as accounting for missing or unknown variables. Table 1 demonstrated that the variables we describe had very few unknown values and thus we do not expect this small number to substantially impact our results. Second, a limitation in identifying our cohort was ensuring that patients who had a diagnosis of prostate cancer received one of the focus drugs for prostate cancer rather than for another diagnosis. Since docetaxel is the only focus drug in our study that is approved for use in other cancers and is typically a salvage treatment in other cancers, we expect that observations of patients using docetaxel for other cancers concurrent with a prostate cancer diagnosis will be uncommon and thus not influence the results substantially. Third, there were limitations in identifying treatment codes since reporting of some treatment codes can be delayed. To handle this limitation, we developed an algorithm based on timing of administration and cost of drug that is explained in our Additional file 1: Supplementary Materials for sample selection. Fourth, the database does not include information about denied claims so it is difficult to know if denials factored into observed patterns of treatments. To address this limitation, we obtained the Coverage Summary and Policy Guidelines for the private insurer which included all of our focus drugs as covered treatments and did not differentiate coverage based on Local Coverage Determinations. Thus, we expect there would not have been a systematic denial of coverage for any of the included treatments and the majority of the geographic variation we detected is likely due to other factors such as patient, provider, or hospital factors, rather than claim denials. We expect that among patients included in OptumInsight who are all insured by the same private insurer, coverage for certain treatments are similar across the country. And finally, another limitation was in regards to generalizing the results of an insured cohort of patients to a broader population of patients, which is an inherent limitation to working with a dataset that is limited to patients who are privately insured. While our results may not be generalizable to uninsured patients, results are likely to be applicable to other commercially insured populations. We plan to conduct future studies to address payer-level variables that affect treatment patterns. Despite these limitations, several research teams have conducted important studies using similar statistical methods to analyze data through the Optum platform, including one study by Hershman et al. investigating socioeconomic factors associated with adherence to breast cancer therapy, [21] and another by Abraham et al. evaluating gastrointestinal bleeding associated with different anticoagulant medications [22].

In future work, we expect to repeat our analyses with longer time of follow-up and to investigate patient and external factors that may influence patterns of treatment, such as the factors that may underlie the geographic variation we observed. Understanding the determinants of this geographic variation is essential to minimizing disparities in treatment delivery. We also expect to repeat this work in broader reaching datasets such as Medicare in order to investigate patterns of care in patients who may not be able to afford private health insurance. As recommendations for using these focus drugs expand, [23, 24] identifying treatment variation and determinants of that variation, geographical or otherwise, become even more important so that implementation of best practices in the use of these treatments across the country can be optimized and tailored to different regions and practices.

## Conclusion

In summary, we were able to identify the patterns of use of novel agents for advanced prostate cancer and demonstrate that abiraterone and enzalutamide have largely replaced intravenous therapy docetaxel as the first-line treatment of advanced prostate cancer. We also observed significant geographic variation in use of treatments for advanced prostate cancer. Describing this current landscape of treatment use is the first step in understanding treatment variation so that our future studies can identify patient and provider characteristics associated with variation and investigate the comparative effectiveness of treatment outcomes in order to identify highest value care.

## Additional file

Additional file 1: Supplementary Materials. Identifying medical claims indicating administration of IV Infusion therapy using OptumInsight. (DOCX 15 kb)

## Abbreviations

ADT: Androgen deprivation therapy
FDA: Food & Drug Administration
mCRPC: metastatic castration-resistant prostate cancer
CDM: Clinformatics Data Mart
OCD: OptumInsight Claims Database
ICD 9-CM: International Statistical Classification of Diseases and Related Health Problems
NDC: National drug codes
HCPCS: Healthcare Common Procedure Coding System
S.D.: Standard deviation
NE: New England: Connecticut, Maine, New Hampshire, Rhode Island, Vermont, Massachusetts
MA: Middle Atlantic: New Jersey, New York, Pennsylvania
SA: South Atlantic: Delaware, Washington D.C., Florida, Georgia, Maryland, North Carolina, South Carolina, Virginia, West Virginia
ENC: East North Central: Illinois, Indiana, Michigan, Ohio, Wisconsin
ESC: East South Central: Alabama, Kentucky, Mississippi, Tennessee
WNC: West North Central: Iowa, Kansas, Minnesota, Missouri, Nebraska, North Dakota, South Dakota
WSC: West South Central: Arkansas, Louisiana, Oklahoma, and Texas
M: Mountain: Arizona, Colorado, Idaho, Montana, Nevada, New Mexico, Utah, Wyoming
PAC: Pacific: Alaska, California, Hawaii, Oregon, Washington
AL: Alabama
AK: Alaska
AZ: Arizona
AK: Arkansas
CA: California
CO: Colorado
CT: Connecticut
DE: Delaware
FL: Florida
GA: Georgia
HI: Hawaii
ID: Idaho
IL: Illinois
IN: Indiana
IA: Iowa
KS: Kansas
KY: Kentucky
LA: Louisiana
MA: Massachusetts
MD: Maryland
MN: Maine
MI: Michigan
MN: Minnesota
MS: Mississippi
MO: Missouri
MT: Montana
NE: Nebraska
NV: Nevada
NH: New Hampshire
NJ: New Jersey
NM: New Mexico
NY: New York
NC: North Carolina
ND: North Dakota
OH: Ohio
OK: Oklahoma
OR: Oregon
PA: Pennsylvania
RI: Rhode Island
SC: South Carolina
SD: South Dakota
TN: Tennessee
TX: Texas
UT: Utah
VT: Vermont
VA: Virginia
WA: Washington
DC: Washington D.C
WV: West Virginia
WI: Wisconsin
WY: Wyoming

## References

[1] Huggins C, Hodges CV (1941). Studies on prostatic cancer. I. The effect of castration, of estrogen and of androgen injection on serum phosphatases in metastatic carcinoma of the prostate. J Cancer Res.

[2] Tannock IF, de Wit R, Berry WR, Horti J, Pluzanska A, Chi K (2004). Docetaxel plus prednisone or mitoxantrone plus prednisone for advanced prostate cancer. N Engl J Med.

[3] Kantoff PW, Higano CS, Shore ND, Berger ER, Small EJ, Penson DF (2010). Sipuleucel-T immunotherapy for castration-resistant prostate cancer. N Engl J Med.

[4] de Bono JS, Oudard S, Ozguroglu M, Hansen S, Machiels JP, Kocak I (2010). Prednisone plus cabazitaxel or mitoxantrone for metastatic castration-resistant prostate cancer progressing after docetaxel treatment: a randomised open-label trial. Lancet.

[5] de Bono JS, Logothetis CJ, Molina A, Fizazi K, North S, Chu L (2011). Abiraterone and increased survival in metastatic prostate cancer. N Engl J Med.

[6] Ryan CJ, Smith MR, de Bono JS, Molina A, Logothetis CJ, de Souza P (2013). Abiraterone in metastatic prostate cancer without previous chemotherapy. N Engl J Med.

[7] Scher HI, Fizazi K, Saad F, Taplin ME, Sternberg CN, Miller K (2012). Increased survival with enzalutamide in prostate cancer after chemotherapy. N Engl J Med.

[8] Beer TM, Armstrong AJ, Rathkopf DE, Loriot Y, Sternberg CN, Higano C (2014). Enzalutamide in metastatic prostate cancer before chemotherapy. N Engl J Med.

[9] Parker C, Nilsson S, Heinrich D, Helle SI, O'Sullivan JM, Fossa SD (2013). Alpha emitter radium-223 and survival in metastatic prostate cancer. N Engl J Med.

[10] Cooperberg MS, Sartor O, Armstrong A, Pieczonka C, Concepcion R, Kassabian V (2014). MP70-02 treatment practice patterns in metastatic castration-resistant prostate cancer patients prior to receiving Sipuleucel-T: data from PROCEED. J Urol.

[11] Caram MEV, Borza T, Min HS, Griggs JJ, Miller DC, Hollenbeck BK (2017). Early National Dissemination of Abiraterone and enzalutamide for advanced prostate cancer in Medicare part D. J Oncol Pract.

[12] McGuire KJ, Harrast J, Herkowitz H, Weinstein JN (2012). Geographic variation in the surgical treatment of degenerative cervical disc disease: American Board of Orthopedic Surgery Quality Improvement Initiative; part II candidates. Spine (Phila Pa 1976).

[13] Boss EF, Marsteller JA, Simon AE (2012). Outpatient tonsillectomy in children: demographic and geographic variation in the United States, 2006. J Pediatr.

[14] Wennberg DE, Kellett MA, Dickens JD, Malenka DJ, Keilson LM, Keller RB (1996). The association between local diagnostic testing intensity and invasive cardiac procedures. JAMA.

[15] Cookson MS, Roth BJ, Dahm P, Engstrom C, Freedland SJ, Hussain M (2013). Castration-resistant prostate cancer: AUA Guideline. J Urol.

[16] Basch E, Loblaw DA, Oliver TK, Carducci M, Chen RC, Frame JN (2014). Systemic therapy in men with metastatic castration-resistant prostate cancer:American Society of Clinical Oncology and Cancer Care Ontario clinical practice guideline. J Clin Oncol.

[17] Hollenbeck BK, Kaufman SR, Yan P, Herrel LA, Borza T, Schroeck FR, et al., Urologist Practice Affiliation and Intensity-modulated Radiation Therapy for Prostate Cancer in the Elderly. Eur Urol, 2017. 10.1016/j.eururo.2017.08.001 PMC5817042 28823605

[18] Zafar SY, Peppercorn JM, Schrag D, Taylor DH, Goetzinger AM, Zhong X (2013). The financial toxicity of cancer treatment: a pilot study assessing out-of-pocket expenses and the insured cancer patient's experience. Oncologist.

[19] Huntington SF (2016). Cancer-related financial toxicity: beyond the realm of drug pricing and out-of-pocket costs. Ann Oncol.

[20] Bestvina CM, Zullig LL, Zafar SY (2014). The implications of out-of-pocket cost of cancer treatment in the USA: a critical appraisal of the literature. Future Oncol.

[21] Hershman DL, Tsui J, Wright JD, Coromilas EJ, Tsai WY, Neugut AI (2015). Household net worth, racial disparities, and hormonal therapy adherence among women with early-stage breast cancer. J Clin Oncol.

[22] Abraham NS, Singh S, Alexander GC, Heien H, Haas LR, Crown W (2015). Comparative risk of gastrointestinal bleeding with dabigatran, rivaroxaban, and warfarin: population based cohort study. BMJ.

[23] Sweeney CJ, Chen YH, Carducci M, Liu G, Jarrard DF, Eisenberger M (2015). Chemohormonal therapy in metastatic hormone-sensitive prostate cancer. N Engl J Med.

[24] Fizazi K, Tran N, Fein L, Matsubara N, Rodriguez-Antolin A, Alekseev BY (2017). Abiraterone plus prednisone in metastatic, castration-sensitive prostate cancer. N Engl J Med.

