# Supplementary material for: Temporal and geographic variation in the systemic treatment of advanced prostate cancer
Source: BMC Cancer. 2018 Mar 6;18:258. doi: 10.1186/s12885-018-4166-3 (PMC5840834; doi:10.1186/s12885-018-4166-3)
Supplement: Supplementary file 1 — Supplementary Materials. Identifying medical claims indicating administration of IV Infusion therapy using OptumInsight. (DOCX 15 kb) [file 12885_2018_4166_MOESM1_ESM.docx]

**Supplementary Materials**

**Identifying medical claims indicating administration of IV Infusion therapy using OptumInsight**

Docetaxel: Match on the first nine-digits of national drug codes (NDCs) and type of service (TOS) codes or Healthcare Common Procedure Coding System (HCPCS) codes and TOS codes.

NDCs:

00069914111, 00069914122, 00069914211, 00069914222, 00069914411, 00075800120, 00075800180, 00075800281, 00075800301, 00075800404, 00075800501, 00409020102, 00409020110, 00409020120, 00409020125, 00409020126, 00409020127, 00703572001, 00703573001, 00955102001, 00955102104, 16714046501, 16714050001, 16729012049, 16729022850, 16729023163, 16729023164, 16729023165, 16729026763, 16729026764, 16729026765, 25021022201, 25021022204, 25021022207, 42367012121, 42367012125, 42367012129, 43598025811, 43598025940, 45963073452, 45963073454, 45963073474, 45963076552, 45963078174, 45963079056, 47335028540, 47335028541, 47335028640, 47335028641, 473350287, 47335028840, 63739093211, 63739097117, 66758005001, 66758005002, 66758005003, 66758095002, 66758095003, 66758095004.

HCPCS codes: J9170, J9171

TOS codes: ANC.DRUGAD

Comments: The NDC codes and HCPCS codes were independently found to be associated with docetaxel (docefrez or taxotere). The claims data was first reduced by matching on NDC then viewing HCPCS codes associated with those records and then the claims data was reduced by matching on HCPCS and viewing the NDC codes associated with those records. No additional relevant NDCs or HCPCS codes were found.

Cabazitaxel**:** Claims data for cabazitaxel may have been documented using a NDC code and/or a HCPCS code, which may or may not be uniquely associated with the therapy. Thus, for each of the years 2010, 2011, 2012, 2013, 2014 and 2015, we implemented the following algorithm.

**2010:** 1) Match on the first nine-digits of independently identified NDCs associated with cabazitaxel (00024582411, 00024582315, 00024582201) and TOS code ANC.DRUGAD. Observe associated HCPCS codes and standard costs that exceed $2000. The only observed HCPCS code is J9999 (not otherwise classified, antineoplastic drugs). 2) Match the claims data against the HCPCS code J9043 known to be uniquely associated with cabazitaxel. No records were found (HCPCS code hasn’t been adopted yet). 3) Match on a) the NDCs 00024582411, 00024582315 or 00024582201 and type of service (TOS) code ANC.DRUGAD or b) HCPCS code J9999 and standard cost observed in (1) and TOS code ANC.DRUGAD. Records satisfying a or b are recorded as administrations of cabazitaxel.

**2011:** 1) Match on the first nine-digits of independently identified NDCs associated with cabazitaxel (00024582411, 00024582315, 00024582201) and TOS code ANC.DRUGAD. Relevant associated HCPCS codes are J9999 (not otherwise classified, antineoplastic drugs), J3490 (unclassified drugs), and J9043. Standard costs that exceed $2000 associated with these claims are recorded. 2) Match the claims data against the HCPCS code J9043 known to be uniquely associated with cabazitaxel. No additional NDC codes are detected. 3) Match on a) the NDCs 00024582411, 00024582315 or 00024582201 and type of service (TOS) code ANC.DRUGAD or b) HCPCS code J9043 and TOS code ANC.DRUGAD or c) HCPCS code J9999 or J3490 and standard cost observed in (1) and TOS code ANC.DRUGAD. Records satisfying a, b or c are recorded as administrations of cabazitaxel.

**2012/2013:** 1) Match on the first nine-digits of independently identified NDCs associated with cabazitaxel (00024582411, 00024582315, 00024582201) and TOS code ANC.DRUGAD. The only relevant associated HCPCS codes are J3490 and J9043. Standard costs that exceed $2000 associated with these claims are recorded. 2) Match the claims data against the HCPCS code J9043 known to be uniquely associated with cabazitaxel. No additional NDC codes are detected. 3) Match on a) the NDCs 00024582411, 00024582315 or 00024582201 and type of service (TOS) code ANC.DRUGAD or b) HCPCS code J9043 and TOS code ANC.DRUGAD or c) HCPCS code J3490 and standard cost observed in (1) and TOS code ANC.DRUGAD. Records satisfying a, b or c are recorded as administrations of cabazitaxel.

**2014/2015:** 1) Match on the first nine-digits of independently identified NDCs associated with cabazitaxel (00024582411, 00024582315, 00024582201) and TOS code ANC.DRUGAD. The only relevant associated HCPCS codes is J9043. 2) Match the claims data against the HCPCS code J9043 known to be uniquely associated with cabazitaxel. No additional NDC codes are detected. 3) Match on a) the NDCs 00024582411, 00024582315 or 00024582201 and type of service (TOS) code ANC.DRUGAD or b) HCPCS code J9043 and TOS code ANC.DRUGAD. Records satisfying a or b are recorded as administrations of cabazitaxel.

Sipuleucel-T**:** Claims data for sipuleucel-T may have been documented using a NDC code and/or a HCPCS code, which may or may not be uniquely associated with the therapy. Thus, for each of the years 2010, 2011, 2012, 2013, 2014 and 2015, we implemented the following algorithm.

**2010:** 1) Match on the first nine-digits of independently identified NDC codes associated with sipuleucel-T (30237890006) and TOS code ANC.DRUGAD. Observe relevant HCPCS codes associated with sipuleucel-T are J3490 and J3590. Standard costs that exceed $5000 associated with these claims are recorded. 2) Match the claims data against the HCPCS code Q2043 or C9273 known to be uniquely associated with sipuleucel-T. No records are obtained (HCPCS codes not yet established). 3) Match on a) the NDC 30237890006 and type of service (TOS) code ANC.DRUGAD or b) HCPCS codes J3490 or J3590 and standard cost observed in (1) and TOS code ANC.DRUGAD. Records satisfying a or b are recorded as administrations of sipuleucel-T.

**2011:** 1) Match on the first nine-digits of independently identified NDC codes associated with sipuleucel-T (30237890006) and TOS code ANC.DRUGAD. Observe relevant HCPCS codes associated with sipuleucel-T are Q2043, J3490, J3590 and J9999. Standard costs that exceed $2000 associated with these claims are recorded. 2) Match the claims data against the HCPCS code Q2043 known to be uniquely associated with sipuleucel-T. No additional NDC codes are detected. 3) Match on a) the NDC 30237890006 and type of service (TOS) code ANC.DRUGAD or b) HCPCS code Q2043 and TOS code ANC.DRUGAD or c) HCPCS codes J3490, J3590 or J9999 and standard cost observed in (1) and TOS code ANC.DRUGAD. Records satisfying a, b or c are recorded as administrations of sipuleucel-T.

**2012/2013/2014/2015:** 1) Match on the first nine-digits of independently identified NDC codes associated with sipuleucel-T (30237890006) and TOS code ANC.DRUGAD. Observe relevant HCPCS code associated with sipuleucel-T is Q2043. 2) Match the claims data against the HCPCS code Q2043 known to be uniquely associated with sipuleucel-T. No additional NDC codes are detected. 3) Match on a) the NDC 30237890006 and type of service (TOS) code ANC.DRUGAD or b) HCPCS code Q2043 and TOS code ANC.DRUGAD. Records satisfying a or b are recorded as administrations of sipuleucel-T.

Radium-223:

**2013/2014:** 1) Match on the first nine-digits of independently identified NDC codes associated with radium-223 (0419020801). Observe relevant HCPCS code associated with radium-223 is A9699 (radiopharmaceutical, therapeutic, not otherwise classified). Standard costs that exceed $1000 associated with these claims are recorded. 2) Match the claims data against the HCPCS code A9606 known to be uniquely associated with radium-223. No records are obtained. 3) Match on a) the NDC 0419020801 or b) HCPCS code A9699 and standard cost observed in (1). Records satisfying a or b are recorded as administrations of radium-223.

**2015:** 1) Match on the first nine-digits of independently identified NDC codes associated with radium-223 (0419020801). Observe relevant HCPCS code associated with radium-223 is A9606. 2) Match the claims data against the HCPCS code A9606 known to be uniquely associated with radium-223. No new NDC codes are detected. 3) Match on a) the NDC 0419020801 or b) HCPCS code A9606. Records satisfying a or b are recorded as administrations of radium-223.
